# Assessing the respective contributions of dietary flavanol monomers and procyanidins in mediating cardiovascular effects in humans: randomized, controlled, double-masked intervention trial

**DOI:** 10.1093/ajcn/nqy229

**Published:** 2018-10-24

**Authors:** Ana Rodriguez-Mateos, Timon Weber, Simon S Skene, Javier I Ottaviani, Alan Crozier, Malte Kelm, Hagen Schroeter, Christian Heiss

**Affiliations:** 1 Division of Cardiology, Pulmonology, and Vascular Medicine, Medical Faculty, University of Dusseldorf, Dusseldorf, Germany; 2 University of Surrey, Faculty of Health and Medical Sciences, Guildford, United Kingdom; 3 Mars, Inc., McLean, VA; 4 Nutrition Department, University of California, Davis, Davis, CA

**Keywords:** cocoa, epicatechin: procyanidins, valerolactones, structurally related (−)-epicatechin metabolites, endothelial function, blood pressure

## Abstract

**Background:**

Flavanols are an important class of food bioactives that can improve vascular function even in healthy subjects. Cocoa flavanols (CFs) are composed principally of the monomer (−)-epicatechin (∼20%), with a degree of polymerisation (DP) of 1 (DP1), and oligomeric procyanidins (∼80%, DP2–10).

**Objective:**

Our objective was to investigate the relative contribution of procyanidins and (−)-epicatechin to CF intake–related improvements in vascular function in healthy volunteers.

**Design:**

In a randomized, controlled, double-masked, parallel-group dietary intervention trial, 45 healthy men (aged 18–35 y) consumed the following once daily for 1 mo: *1*) a DP1–10 cocoa extract containing 130 mg (−)-epicatechin and 560 mg procyanidins, *2*) a DP2–10 cocoa extract containing 20 mg (−)-epicatechin and 540 mg procyanidins, or *3*) a control capsule, which was flavanol-free but had identical micro- and macronutrient composition.

**Results:**

Consumption of DP1–10, but not of either DP2–10 or the control capsule, significantly increased flow-mediated vasodilation (primary endpoint) and the concentration of structurally related (−)-epicatechin metabolites (SREMs) in the circulatory system while decreasing pulse wave velocity and blood pressure. Total cholesterol significantly decreased after daily intake of both DP1–10 and DP2–10 as compared with the control.

**Conclusions:**

CF-related improvements in vascular function are predominantly related to the intake of flavanol monomers and circulating SREMs in healthy humans but not to the more abundant procyanidins and gut microbiome–derived CF catabolites. Reduction in total cholesterol was linked to consumption of procyanidins but not necessarily to that of (−)-epicatechin. This trial was registered at clinicaltrials.gov as NCT02728466.

## INTRODUCTION

Evidence from clinical dietary intervention studies has accumulated that links cocoa flavanol (CF) intake in humans with improvements in vascular function and health (1). CFs comprise several individual compounds, including monomeric flavanols such as (–)-epicatechin and typically smaller amounts of (+)-catechin, as well as procyanidins, which represent oligomeric flavanol derivatives with a degree of polymerization (DP) ranging from 2 to 10 (2). Whereas (–)-epicatechin can represent between 15% and 20% (by wt) of CFs, procyanidins make up 80–85% (by wt) (3).

**FIGURE 1:**
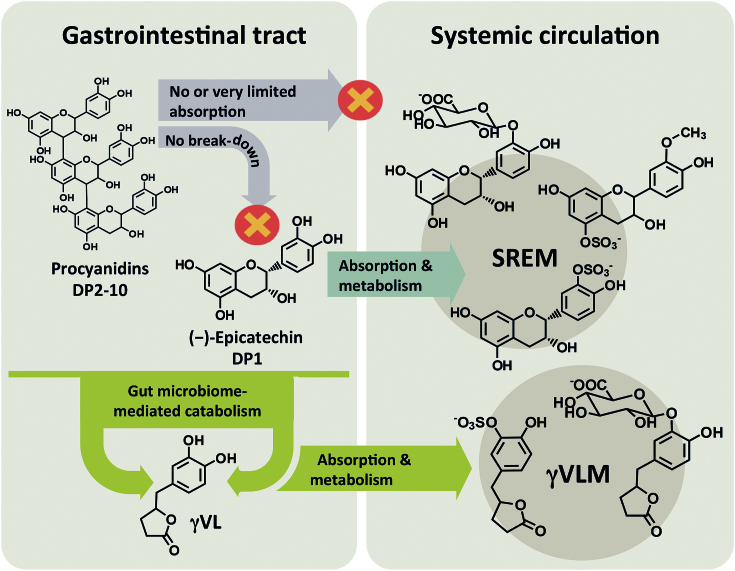
Schematic of the fate of cocoa flavanols in the gastrointestinal tract. Only the monomeric flavanol (−)-epicatechin is absorbed in the small intestine and metabolized to the major SREMs (–)-epicatechin-3′-*O*-glucuronide, 3′-*O*-methyl-(–)-epicatechin-5-sulfate, and (–)-epicatechin-3′-sufate. Oligomeric procyanidins are neither absorbed nor metabolized in the small intestine. Both monomers and procyanidins are catabolized by gut microbes in the colon, leading to ring fission products including γVL, which, in turn, is absorbed and further metabolized to γVLMs. DP, degree of polymerization; SREM, structurally related (−)-epicatechin metabolite; γVL, 5-(3′,4′-dihydroxyphenyl)-γ-valerolactone; γVLM, phase II γVL metabolite.

We have previously established a causality chain between the intake of one of the constituents of CF, namely (−)-epicatechin, the subsequent absorption and presence of (−)-epicatechin metabolites in the systemic circulation, and acute nitric oxide–dependent arterial dilation [i.e., flow-mediated vasodilation (FMD)] in humans (4). In this context, we and others have shown that (−)-epicatechin is absorbed in the proximal gastrointestinal tract (GIT) and is transiently present in the human systemic circulation in the form of various structurally related (−)-epicatechin metabolites (SREMs), which include (–)-epicatechin-3′-*O*-glucuronide, 3′-*O*-methyl-(–)-epicatechin-5- and -7-sulfate, and (–)-epicatechin-3′-sulfate (5, 6) (**Figure 1**).

In contrast, there is no evidence that procyanidins, especially those with a DP of >2, are absorbed in the GIT as intact procyanidin molecules, and they are not present in the systemic circulation in humans (7). Nor do procyanidins break down in the GIT in ways that contribute to the circulating pool of SREMs (7). However, novel advances demonstrate that the human microbiome may play a crucial role in understanding the bioactivity of CFs (6). Approximately 70% of the ingested (−)-epicatechin is absorbed via the colon after catabolism by the microbiota, giving rise in the systemic circulation to sulfated and glucuronidated metabolites of 5-(3′,4′-dihydroxyphenyl)-γ-valerolactone (γVL) and 5-(3′,4′-dihydroxyphenyl)-γ-hydroxyvaleric acid, corresponding to 42% ± 5% of (−)-epicatechin intake (6). Procyanidins are also subject to microbiome-mediated catabolism, which contributes significantly to the concentrations of γVL metabolites in humans (7, 8). Overall, we estimated that 43% and 54% of the γVL metabolites present in human plasma after the intake of CFs originate from the flavanol monomers and procyanidins, respectively (7). Taken together, the role of the gut microbiome in flavanol and procyanidin absorption and metabolism is important, because gut microbiome–derived CF metabolites represent a major part of the CF metabolome present in the human circulation. Thus, γVL metabolites represent principal candidates for CF-derived metabolites causal in mediating the cardiovascular effects observed after dietary CF intake. Consequently, with the use of CF as a model, the main goal of this investigation was to undertake a dietary intervention study to establish the respective relative contributions of flavanol monomers and DP2–10 procyanidins in mediating the cardiovascular effects observed after cocoa intake in humans and to assess the roles of SREMs and γVL metabolites in these processes.

## METHODS

### Study subjects

**FIGURE 2:**
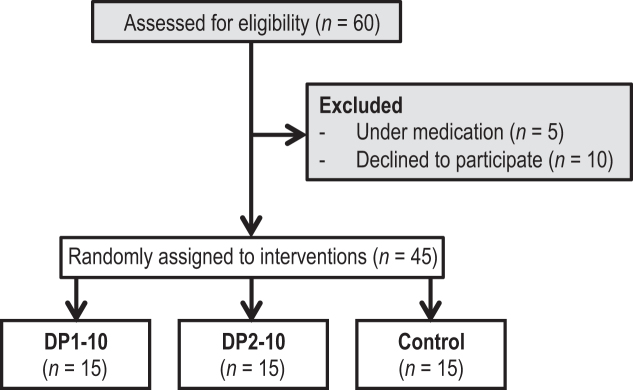
CONSORT study flow. CONSORT, Consolidated Standards of Reporting Trials; DP, degree of polymerization.

A total of 45 healthy Caucasian men, aged between 18 and 35 y, were recruited at the University of Dusseldorf (January 2015–May 2016). [See **Table 1** for their characteristics and **Figure 2** for the Consolidated Standards of Reporting Trials (CONSORT) study flow]. The study subjects were screened based on a clinical physical examination that included blood pressure measurement, an electrocardiogram, and routine clinical tests (blood lipids, C-reactive protein, full blood count, liver enzymes, hemoglobin, glucose). Inclusion criteria for participation in the study were as follows: 18–35 y of age, Caucasian, male, BMI (kg/m^2^) of 23–27, and signed consent form. Exclusion criteria were clinical signs of symptoms of manifest cardiovascular disease (coronary artery disease, peripheral artery disease, cardiovascular disease), diabetes, heart rhythm other than sinus rhythm, allergy to milk products, or sensitivity to the methylxanthines, caffeine, and theobromine. We also excluded subjects who had taken antibiotics or vitamin supplements within the previous 3 mo, were on an extreme diet (vegan, vegetarian), or had consumed >2 alcoholic drinks/d (>210 g alcohol/wk).

**TABLE 1:** Baseline characteristics of the study population^1^

	DP1–10	DP2–10	Control
*n*	15	15	15
Age, y	23 ± 2	25 ± 2	23 ± 2
Weight, kg	78 ± 8	79 ± 10	79 ± 10
Height, m	1.81 ± 0.05	1.81 ± 0.07	1.85 ± 0.07
BMI, kg/m^2^	23.6 ± 0.5	24.1 ± 2.2	23.1 ± 2.4
Smoker, *n*	0	3	5
Exercise, h/wk	3.2 ± 1.4	2.1 ± 1.2	2.7 ± 1.3
Alcohol, g/wk	89 ± 43	88 ± 49	97 ± 41
Vegetarian, *n*	0	0	1
Creatinine, mg/dL	0.9 ± 0.1	0.9 ± 0.1	0.9 ± 0.1
Bilirubin, mg/dL	0.9 ± 0.6	0.7 ± 0.3	1.1 ± 1
Urate, mg/dL	5.3 ± 1.2	5.9 ± 1.0	5.8 ± 0.9
GGT, mg/dL	30 ± 26	28 ± 31	33 ± 14
GPT, mg/dL	32 ± 34	30 ± 23	32 ± 15
GOT, mg/dL	28 ± 12	37 ± 30	45 ± 37
Heart rate, bpm	65 ± 6	69 ± 8	70 ± 6

^1^Values are means ± SDs unless otherwise indicated. bpm, beats per minute; DP, degree of polymerization; GGT, γ-glutamyltransferase; GOT, glutamate oxaloacetate transaminase; GPT, glutamyl pyruvate transferase.

### Test materials

All test materials were provided by Mars, Inc., in the form of identical capsules that were in identical bottles, each labeled with 1 of 3 alpha-numeric codes. Each participant consumed 2 capsules/d. The DP1–10 capsules made with cocoa extract provided 690 mg total CFs, of which 130 mg was (−)-epicatechin and 560 mg was DP2–10 procyanidins. The DP2–10 capsules contained a cocoa extract with total of 560 mg CFs, of which 20 mg was (−)-epicatechin and 540 mg was DP2–10 procyanidins. The control capsules contained no CFs, but their caffeine and theobromine content was matched to that of the DP1–10 and DP2–10 capsules (**Table 2**).

**TABLE 2:** Composition of the test interventions^1^

	DP1–10	DP2–10	Control
Total cocoa flavanols, mg	690	560	0
(−)-Epicatechin, mg	130	20	0
Dimers-decamers, mg	560	540	0
Theobromine, mg	80	80	80
Caffeine, mg	20	20	20

^1^Values reflect daily ingested amount and were delivered in 2 capsules. DP, degree of polymerization.

### Study design

A randomized, controlled, double-masked, and parallel-group dietary intervention trial was performed consisting of 3 interventions. The 45 study participants were assigned to 1 of 3 parallel groups (*n* = 15 each): the DP1–10, DP2–10, or control group (Table 2). The randomized assignment list was generated with a software freely available on www.graphpad.com/quickcalcs/randomize2/ (GraphPad Software, Inc.).

Each intervention was preceded by a 24-h low-flavonoid diet, where volunteers were instructed to refrain from the consumption of flavonoid-rich foods, including fruits and vegetables, cocoa, chocolate, tea, coffee, and alcohol. During each arm, participants consumed daily with their breakfast, for a period of 1 mo, 2 capsules containing one of *1*) DP1–10 flavanol monomers and procyanidins,*2*) DP2–10 procyanidins, or *3*) a flavanol-free control extract with matched micro- and macronutrient composition (Table 2).

Measurements were taken before (baseline) and at 2 h after the first capsules on day 1 and at 1 mo (days 28–30). Compliance was assessed by the collection of empty bottles on the last study day visit.

FMD was the primary endpoint. Secondary endpoints were plasma and urinary concentrations of SREMs and sulfated and glucuronidated metabolites of the colonic catabolite γVL. Tertiary endpoints included other determinants of vascular function, such as blood pressure, arterial stiffness [including pulse wave velocity (PWV) and augmentation index], blood lipids (plasma concentrations of total cholesterol, HDL cholesterol, LDL cholesterol, and triglycerides ), and fasting plasma glucose.

Volunteers were asked to follow a low-flavonoid diet until the next morning when the 24-h urine was collected. Volunteers were also asked to complete a 48-h dietary questionnaire to assess compliance with the low-flavonoid diet. A qualified researcher enrolled the participants into the study. All researchers involved in conducting the study or assessing outcomes were blinded with regard to the identity of the test products. The study was conducted according to the guidelines of the Declaration of Helsinki, all procedures involving human subjects were approved by the University of Dusseldorf Research Ethics Committee, and written informed consent was obtained from all study participants. The study was registered with the NIH randomized trials registry at clinicaltrials.gov as NCT02728466.

### FMD and power analysis of the primary endpoint

FMD was measured in the brachial artery of the right arm by the use of 5-min forearm occlusion by ultrasound (Vivid I; GE Healthcare) in combination with a semiautomated analysis system (Brachial Analyzer, MIA) (9). The intra- and interindividual variabilities in FMD measurements established in our laboratory are 0.9% (the SD of the difference between repeated FMD measurements in *n* = 20 healthy subjects; Christian Heiss, 2015) and 1% (the SD within a group of healthy subjects), respectively (10). FMD was defined as the primary outcome. Based on previous intervention studies with CFs, a change in FMD of 1.3% was anticipated (11). Assuming an SD of the change in FMD of 1%, 15 subjects/group would provide sufficient power to detect an absolute change in FMD of 1.2% (2-sided α of 0.0167%, power = 0.80) between groups with the use of a Bonferroni correction in pairwise comparisons (12).

### Quantification of SREMs and γVL metabolites by HPLC

SREMs and γVL metabolites were analyzed in urine and plasma by HPLC with fluorescence detection, as described by Ottaviani et al. (5, 7). Blood samples were collected before and at 2 h postconsumption on day 1 and after 1 mo of daily consumption of test capsules with the use of EDTA-coated tubes. Plasma was obtained by whole-blood centrifugation at 1800 × *g* for 15 min at 4°C, separated into several 1.2-mL aliquots and spiked with ascorbic acid to provide a final concentration of 1 mg/mL. Subjects were also asked to collect urine samples for 24 h following test product consumption on day 1 and at 1 mo. The total volume for each period was recorded. Immediately after collection, urine samples were transferred into 14-mL tubes, acidified with 560 µL of 2 mol sodium acetate/L at pH 4.5 and 56 µL of 0.5% thymol (wt:vol) in isopropanol, and finally stored at −80°C prior to analysis.

### Blood pressure measurements

Office blood pressure was measured with the use of an automated clinical digital sphygmomanometer (Dynamap) at the upper left arm in a supine position, after 10 min of supine rest in a quiet room and with the arm at heart level. At each time point, 3 blood pressure measurements were taken. The first was discarded and the remaining 2 were used to provide an average that was used for further analyses.

### PWV

PWV was determined from applanation tonometry measurements taken at the carotid and femoral arteries with a SphygmoCor system, as previously described (13).

### Blood lipids and glucose measurements

Total cholesterol, HDL cholesterol, LDL cholesterol, triglycerides, and fasting plasma glucose were measured by the Institute for Clinical Chemistry, University of Dusseldorf, with the use of standard accredited assays.

### Statistical analysis

The characteristics of the study population are expressed as means ± SDs (Table 1). The primary test for an effect in the randomized controlled trial was a univariate ANCOVA followed by post hoc pairwise comparisons comparing the responses due to DP1–10, DP2–10, and the control (fixed factors) at 1 mo (dependent) with baseline values as covariates to account for baseline differences. Responses to treatments were calculated as changes in respective parameters (e.g., FMD): 1-mo values minus baseline values on day 1. Mean values of parameters are presented as means ± SEMs, and differences between responses are presented as means with Bonferroni-adjusted 95% CIs. We also analyzed the difference between responses at 2 h after acute consumption of the 3 interventions on day 1 and at 1 mo as compared with the 0-h baseline on day 1 by the use of repeated-measurements ANCOVA, with baseline values as covariates. Analyses were computed with SPSS 24 (IBM Corp.).

## RESULTS

The study population consisted of 45 young, healthy male volunteers (Figure 2), and their baseline characteristics are detailed in Table 1. All values were within the normal range for healthy individuals, and there were no significant differences between groups. The test capsules were well tolerated, and no adverse events were reported in the context of this study. All subjects assigned to the interventions received the interventions, completed the trial, and were analyzed for the primary endpoint.

**FIGURE 3:**
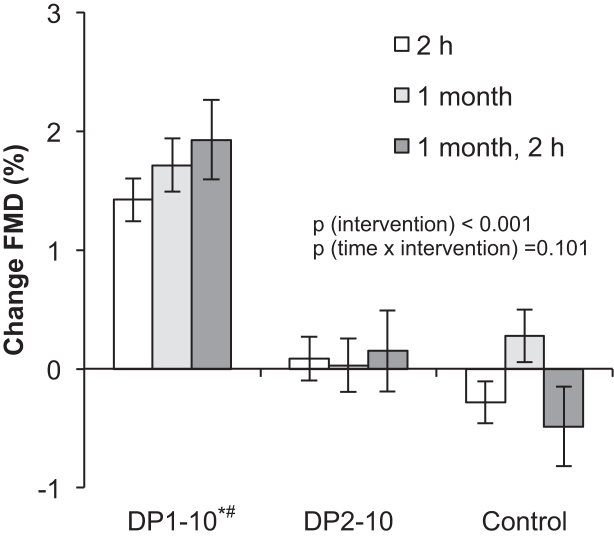
Changes in values of FMD from baseline at 2 h, 1 mo, and 2 h at 1 mo after supplementation of the following: *1*) DP1–10, standardized cocoa extract that contains monomeric flavanols, mainly (−)-epicatechin, and procyanidins with a DP that ranges from 2 to 10; *2*) a cocoa extract that contains predominantly procyanidins (DP2–10); or *3*) a flavanol-free control (Control) (see Table 1 for their compositions). Time × intervention interaction, *P* = 0.101; main effect of intervention, *P* < 0.001; repeated-measurements ANCOVA with baseline values as covariates with Bonferroni post hoc test. Values are means ± SEMs. *P < 0.05 vs. DP2–10. ^#^*P* < 0.05 vs. Control. DP, degree of polymerization; FMD, flow-mediated vasodilation.

### Endothelial function improves after intake of DP1–10 flavanols but not after intake of DP2–10 flavanols

During this 1-mo randomized controlled trial, the consumption of DP1–10 led to a significant improvement in FMD as compared with the control and DP2–10 (**Table 3**), taking baseline values of FMD as a covariate into account. FMD improvement after the consumption of DP1–10 over the control at 1 mo was 1.4% (95% CI: 0.7%, 2.2%). FMD improvement after the consumption of DP1–10 over DP2–10 at 1 mo was 1.7% (95% CI: 0.9%, 2.5%). **Figure 3** shows that similar improvements were observed at 2 h after the first capsules on day 1 but that there were no further improvements at 1 mo and 2 h after consumption of the last DP1–10 capsules, but not after taking DP2–10 or the control capsules. There was no significant time × intervention interaction (*P* = 0.101), suggesting that the effect of DP1–10 did not differ between the time points. The baseline diameter of the brachial artery and endothelium-independent nitroglycerin-mediated vasodilation remained unaffected by either intervention (data not shown).

**TABLE 3:** Overview of endpoints at baseline and after daily consumption of DP1–10 (containing flavanol monomers and procyanidins), DP2–10 (containing procyanidins), or control capsules for 1 mo and differences between changes in endpoints^1^

	DP1–10 (*n* = 15)		DP2–10 (*n* = 15)		Control (*n* = 15)		Difference at 1 mo			
	Baseline	1 mo	Baseline	1 mo	Baseline	1 mo	DP1–10 vs. control	DP2–10 vs. control	DP1–10 vs. DP2–10	*P* ^2^
Primary endpoint										
FMD, %	6.4 ± 0.4	8.2 ± 0.3	7.3 ± 0.4	7.2 ± 0.5	6.5 ± 0.4	6.9 ± 0.3	1.4 (0.7, 2.2)*	−0.2 (−1.0, 0.6)	1.7 (0.9, 2.5)*	<0.001
Secondary endpoints										
Plasma SREMs, nmol/L	2 ± 2	86 ± 52	2 ± 2	4 ± 3	0 ± 0	7 ± 7	48 (−26, 123)	−26 (−101, 48)	75 (1, 148)*	0.048
Plasma γVLs, nmol/L	55 ± 14	150 ± 34	26 ± 5	76 ± 15	46 ± 10	55 ± 8	94 (15, 174)*	22 (−58, 103)	72 (−10, 155)	0.014
Tertiary endpoint										
Office SBP, mm Hg	134 ± 3	125 ± 2	126 ± 2	126 ± 2	126 ± 2	126 ± 2	−6.7 (−12.6, −0.9)*	−0.3 (−5.8, 5.3)	−6.5 (−12.4, −0.6)*	0.010
Office DBP, mm Hg	78 ± 2	70 ± 2	73 ± 2	75 ± 2	73 ± 2	74 ± 2	−5.5 (−11.6, 0.6)	3.2 (−4.8, 11.1)	−6.9 (−13.0, −0.8)*	0.020
PWV, m/s	5.7 ± 0.2	5.3 ± 0.2	6.0 ± 0.2	5.6 ± 0.2	5.4 ± 0.1	5.6 ± 0.3	−1.0 (−1.6, −0.4)*	−0.1 (−0.7, 0.5)	−0.8 (−1.4, −0.2)*	0.001
Total cholesterol, mg/dL	177 ± 12	163 ± 12	170 ± 7	160 ± 6	173 ± 7	182 ± 7	−22 (−40, −4)*	−19 (−37, −1)*	−3 (−20, 15)	0.007
HDL cholesterol, mg/dL	65 ± 4	59 ± 4	60 ± 2	56 ± 2	61 ± 2	62 ± 3	−6 (−13, 1)	−5 (−12, 2)	−1 (−9, 6)	0.114
LDL cholesterol, mg/dL	100 ± 14	100 ± 11	107 ± 9	100 ± 5	113 ± 8	109 ± 8	−15 (−34, 4)	−15 (−35, 4)	0 (−17, 18)	0.102
Triglycerides, mg/dL	121 ± 25	108 ± 23	71 ± 7	82 ± 8	109 ± 18	113 ± 13	−24 (−64, 16)	−11 (−50, 27)	−13 (−54, −29)	0.330
Glucose, mg/dL	83 ± 2	77 ± 2	81 ± 1	81 ± 1	80 ± 3	80 ± 2	−1 (7, 4)	2 (−3, 7)	−3 (−9, 2)	0.247

^1^Values are means ± SEMs or means (Bonferroni-corrected 95% CIs). *Significant. DBP, diastolic blood pressure; DP, degree of polymerization; FMD, flow-mediated vasodilation; PWV, pulse wave velocity; SBP, systolic blood pressure; SREM, structurally related epicatechin metabolite; γVL, valerolactone metabolite.

^2^Derived by using 1-factor ANCOVA with baseline value as covariate.

### SREMs and γVL metabolites in plasma and urine

**FIGURE 4:**
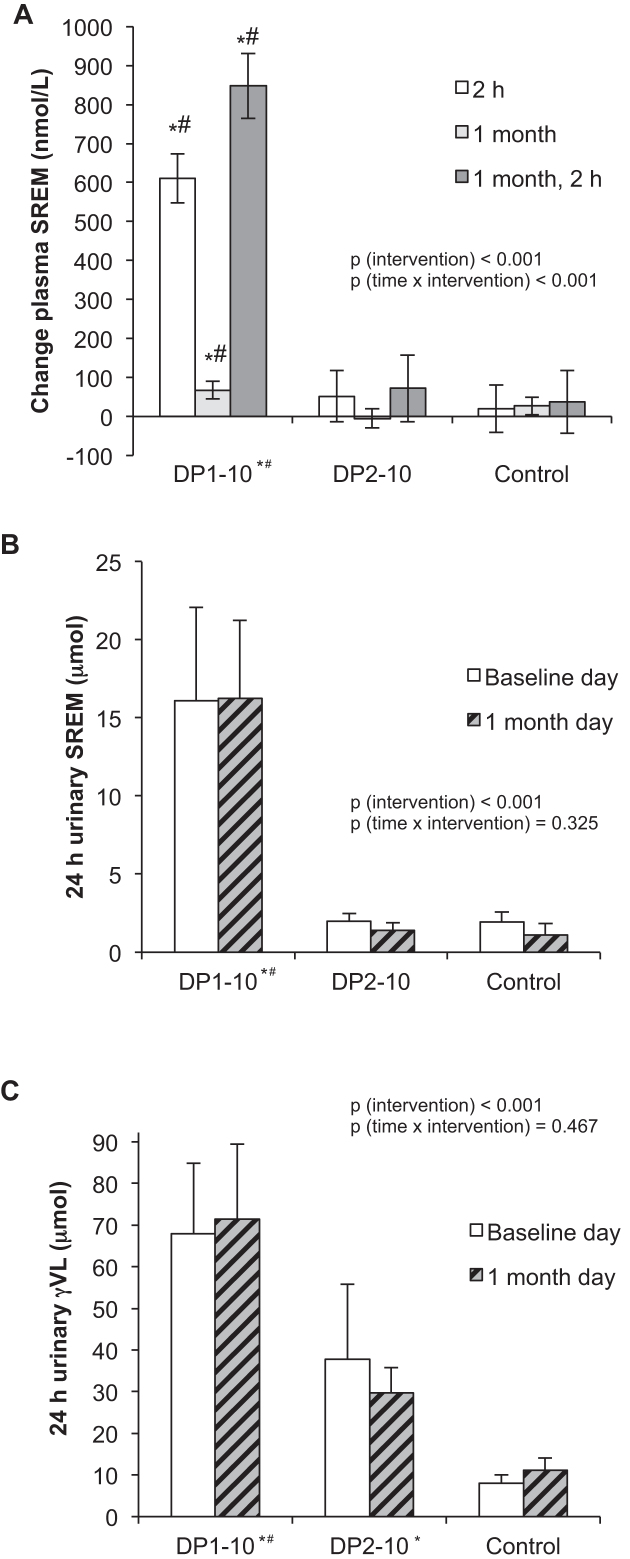
(A) Changes in plasma SREMs from baseline at 2 h, 1 mo, and 2 h at 1 mo after supplementation of the following: *1*) DP1–10, a standardized cocoa extract that contains monomeric flavanols, mainly (−)-epicatechin, and procyanidins with a DP that ranges from 2 to 10; *2*) a cocoa extract that contains predominantly procyanidins (DP2–10); or *3*) a flavanol-free control (Control) (see Table 1 for their compositions). Time × intervention interaction, *P* < 0.001; main effect of intervention, *P* < 0.001; repeated-measurements ANCOVA with baseline values as covariates with Bonferroni post hoc test. Amount of SREMs (B) and γVL metabolites (C) excreted over 24 h in urine on day 1 and the last day of study at 1 mo (time by intervention interactions *P* = 0.325 and *P* = 0.467 and main effects of intervention, each *P* < 0.001; repeated-measurements ANOVA with Bonferroni post hoc test). All values are means ± SEMs. *P < 0.05 vs. DP2–10; ^#^*P* < 0.05 vs. Control. DP, degree of polymerization; SREM, structurally related (−)-epicatechin metabolite; γVL, 5-(3′,4′-dihydroxyphenyl)-γ-valerolactone.

At the beginning of the study on day 1, the concentrations of SREMs in all but 2 subjects were below the limit of detection (Table 3), which is consistent with the low-flavonoid diet that volunteers followed for 24 h prior to the beginning of the study. Ingestion of DP1–10, but not DP2–10 or the control, led to a significant increase in plasma SREMs by 611 nmol/L (95% CI: 483, 739 nmol/L; **Figure 4**A) at 2 h after acute consumption on day 1. After 1 mo of daily consumption and following overnight fasting, fasting concentrations were low and not significantly different from control or DP2–10 concentrations. These results could be explained by the short half-life of SREMs and the overnight fasting of the study subjects prior to the measurements (8). In contrast to the control and DP2–10, additional acute ingestion of DP1–10 led to an increase of 847 nmol/L (95% CI: 679, 1016 nmol/L) in the concentration of plasma SREMs. However, these concentrations were not significantly different from the increases observed after the acute intake on day 1.

Analysis of 24-h urine (Figure 4B) showed that only DP1–10, but not DP2–10, led to significantly higher urinary excretion of SREMs compared with the control, with no difference between day 1 and 1 mo. Although γVL metabolites result from gut microbiome cleavage of both flavanol monomers and procyanidins, cleavage is more efficient in monomers (8). Plasma concentrations of γVL metabolites were only significantly increased at 1 mo in the DP1–10 group; the increase after DP2–10 consumption was not significant (Table 3). However, the analysis of 24-h urine (Figure 4C) showed that the urinary excretion of γVL metabolites was significantly greater in both DP1–10 and DP2–10 compared with the control, with no difference between day 1 and 1 mo. However, DP1–10 led to significantly greater amounts of excreted γVL metabolites.

### Intake of DP1-10 flavanols, but not DP2-10, decreases blood pressure and PWV

**FIGURE 5:**
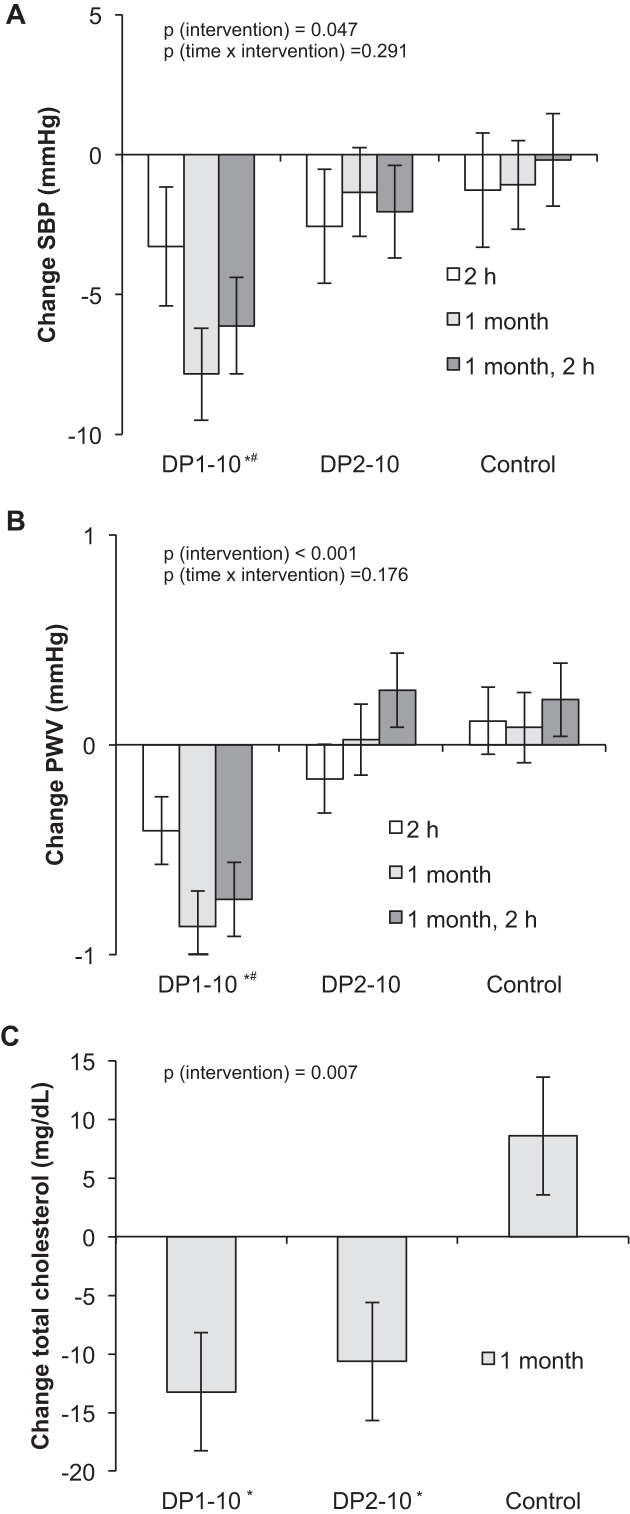
Changes in SBP (A) and PWV (B) from baseline at 2 h, 1 mo, and 2 h at 1 mo and total cholesterol (C) at 1 mo after supplementation of the following: *1*) DP1–10, a standardized cocoa extract that contains monomeric flavanols, mainly (−)-epicatechin, and procyanidins with a DP that ranges from 2 to 10; *2*) a cocoa extract that contains predominantly procyanidins (DP2–10); or *3*) a flavanol-free control (Control) (see Table 1 for their compositions). Repeated-measurements ANCOVA (A, B) and univariate ANCOVA (C), with baseline values as covariates with Bonferroni post hoc test; time × intervention interactions, *P* = 0.291 (A) and *P* = 0.176 (B); main effects of intervention, *P* = 0.047 (A), *P* < 0.001 (B), and *P* = 0.007 (C) .Values are means ± SEMs. *P < 0.05 vs. DP2–10; ^#^*P* < 0.05 vs. Control. DP, degree of polymerization; PWV, pulse wave velocity; SBP, systolic blood pressure.

A significant decrease in office systolic blood pressure was observed at 1 mo after consumption of DP1–10 compared with the control (−6.7 mm Hg; 95% CI: –12.6, –0.9 mm Hg) and DP2–10 (−6.5 mm Hg; 95% CI: –12.4, –0.6 mm Hg). No changes in systolic blood pressure after DP2–10 consumption were seen with respect to the control (−0.3 mm Hg; 95% CI: –5.8, 5.3 mm Hg). The time × intervention interaction was not significant (*P* = 0.271), suggesting that the effect of DP1–10 did not differ between the time points (**Figure 5**A).

A decrease in office diastolic blood pressure was also observed 1 mo after consumption of DP1–10 as compared with DP2–10 (–6.9 mm Hg; 95% CI: –13.0, –0.8 mm Hg), but was not significant when compared with the control (−5.5 mm Hg; 95% CI: –11.6, –0.6 mm Hg), taking the baseline diastolic blood pressure as a covariate into account. The consumption of DP1–10 led to a significant decrease in PWV at 1 mo of −1.0 m/s (95% CI: −1.6, −0.4 m/s) compared with the control and of −0.8 m/s (95% CI: −1.4, −0.2 m/s) compared with DP2–10 (Table 3, Figure 5B).

### Blood lipids and glucose: both DP1-10 and DP2-10 decrease total cholesterol

Both DP1–10 (–22 mg/dL; 95% CI: –40, –4 mg/dL) and DP2–10 (–19 mg/dL; 95% CI: –37, –1 mg/dL) led to a significant decrease in total cholesterol after 1 mo of consumption compared with the control (Figure 5C). No significant changes in triglycerides, plasma glucose, or HDL or LDL cholesterol were observed in any of the groups.

## DISCUSSION

With the use of CFs as a model, the relative contribution of flavanol monomers and procyanidins in mediating the cardiovascular effects observed after dietary intake was assessed. It was also our intention to gain insights into the potential role of gut microbiome–derived CF metabolites in mediating the bioactivity of flavanol monomers and procyanidins. The data obtained show that acute and sustained intake of CF DP1–10, containing 130 mg of the monomer (−)-epicatechin and 560 mg of procyanidins, led to significant improvements in endothelial function and decreases in blood pressure and vascular stiffness. In contrast, intake of DP2–10, which contained 540 mg of procyanidins and a much-reduced 20 mg of (−)-epicatechin, had no significant effect on these parameters.

Based on experiments performed in vitro, it was previously speculated that, upon ingestion, dietary procyanidins are broken down by the colonic microbiome to yield (−)-epicatechin or other flavanol monomers (14). However, in humans in vivo, procyanidin digestion does not yield (−)-epicatechin, and consequently procyanidins do not contribute to the circulating pool of SREMs (7). Instead, after intake, procyanidins are degraded by the colonic microbiota, and the resulting catabolites, including γVLs, are absorbed in the distal GIT (7). The current investigation confirmed these findings and demonstrated that the consumption of CF procyanidins DP2–10 resulted in significant increases in γVL metabolites, but not SREMs, in plasma and urine. This demonstrates that the impact of CF on vascular health is linked to (−)-epicatechin and not to CF procyanidins, and it follows that the vascular effects on FMD, PWV, and blood pressure are mediated by SREMs and not γVL metabolites.

Based on the 30-d duration of the study and the range of the outcomes investigated, it is tenable that significant direct effects of procyanidin intake and γVL metabolites on vascular function would have been observable if γVL metabolites exhibited bioactivity in this context. Although we consider this interpretation as relevant and likely applicable at a larger scale, in view of the inherent limitations of the study design, and in particular the relatively small number of subjects, we cannot completely rule out the possibility that in longer-term exposures of larger groups of subjects who are more representative of the general population circulating γVL metabolites may be demonstrated to exert effects on cardiovascular function. Furthermore, this investigation did not extend to potential procyanidin intake–related and γVL metabolite–mediated health benefits beyond the modulation of vascular function. In addition, circulating γVLs represent a recently validated nutritional biomarker of flavanol monomer and procyanidin intake (8), and thus continue to be of great interest in this area. This overall conclusion supports the notion that SREMs are the bioactive mediators of CF intake–related vascular effects and provides greater clarity and guidance for investigations, both in vitro and ex vivo, that are aimed at elucidating the specific mechanisms of action underlying the biological effects observed after the intake of CFs. These findings also have important implications beyond CFs, inasfar as they are very likely applicable to other flavanol-containing foodstuffs. However, this does not mean that procyanidin intake is without any impact on cardiovascular health, and although not directly affecting FMD, PWV, or blood pressure, procyanidins may affect the effect size of flavanol- or SREM-mediated effects by preserving and protecting flavanols, such as (−)-epicatechin, from oxidation or degradation in foods during processing and storage or in the GIT during digestion. Moreover, procyanidin intake may, via its impact on cholesterol absorption and fecal steroid excretion inside the GIT, mediate decreases in total plasma cholesterol concentrations, as seen here in humans (Table 3) and elsewhere in rats (15), and thus exert cardiovascular health benefits over greater time spans than could be investigated in the current study. The latter 2 points are also highly relevant in the context of 2 recent publications that aimed at assessing the biological effects of pure epicatechin in the context of cardiovascular function, and which did not find significant effects (16–18). When designing studies that use pure epicatechin, based on replicating previous investigations that use epicatechin-containing foods or food extracts, such as cocoa or cocoa extracts, various aspects should be taken into account. Not just the epicatechin intake amount studied (19, 20) but also the composition of the food matrix in which the compound is ingested have to be considered. In this context, the various indirect effects of procyanidins described above and elsewhere, as well as the effect of size-modulating coingestion of methylxanthines (caffeine and/or theobromine) (19) naturally present in cocoa and tea, are important factors in the effect size as it relates to the cardiovascular effects thus far investigated. Consequently, direct comparisons between the effects of epicatechin ingested as an isolated compound and consumed as part of a complex food matrix need to be considered carefully.

Although research into the role of the gut microbiome in human health is still in its infancy, especially in the context of nutrition and primary disease prevention, various reports highlight the importance of specific members of the microbiome or specific microbiome-derived metabolites for human health (21–24). Although a specific analysis of the gut microbiome and its constituent phyla and genera lay outside the scope of this investigation, the specific γVL metabolites identified here in the context of flavanol and procyanidin intake were present in all participants of this study. Further, the work of previous investigators also supports the supposition that the gut microbiome–mediated formation of γVLs is more likely than not to be an event common across human populations and a wider range of diet and lifestyle choices (6). Although the biological effects of circulating γVL metabolites, if any, are currently unknown, data based on testing these compounds in cell culture models suggest various potential bioactivities (25, 26). Whether or not these in vitro findings can be translated into the context of human health maintenance has yet to be established.

In conclusion, our data indicate that the vascular effects after CF intake can be predominantly ascribed to the bioactivity of (−)-epicatechin and that procyanidins do not contribute directly to CF intake–mediated acute and longer-term vascular effects in healthy humans. In view of potential future dietary recommendations for flavanol intake in the context of cardiovascular health maintenance and disease prevention, these findings are of great relevance. Further large-scale research is needed to confirm or refute this notion, but ongoing studies, especially the 5-y COSMOS trial, which is currently studying the effects of CF intake on mortality and cardiovascular morbidity in 22,000 men and women in the United States, will be important for doing so. Moreover, in order to be able to establish recommended intake values or to recommend the consumption of certain foods, the biologically active compounds should be identified and their content in foods should be known. Although considerable efforts have been put into the establishment of the Phenol Explorer and other food content databases (27), our general understanding of flavanol and polyphenol intake is still limited. Also, the common approach of estimating intake by combining food intake data with food content databases is severely limited and not currently validated by independent (e.g., biomarker-based) means (28). The current study further underscores that a more detailed, accurate, quantitative knowledge of individual (poly)phenols is of importance in predicting potential biological effects of foods and, thus, in the development of evidence-based nutritional recommendations.

## Notes

Funding was provided through an unrestricted grant by Mars, Inc. Mars, Inc., also provided the standardized test capsules used in this investigation.

Present address for ARM: Department of Nutritional Sciences, School of Life Course Sciences, Faculty of Life Sciences and Medicine, King's College London, London, United Kingdom.

Present address for CH: University of Surrey, Faculty of Health and Medical Sciences, Guildford, United Kingdom and Surrey and Sussex Healthcare NHS Trust, Redhill, United Kingdom.

HS and CH contributed equally to this work.

Abbreviations used:

CF: cocoa flavanol
DP: degree of polymerization
FMD: flow-mediated vasodilation
GIT: gastrointestinal tract
PWV: pulse wave velocity
SREM: structurally related (−)-epicatechin metabolite
γVL: 5-(3′,4′-dihydroxyphenyl)-γ-valerolactone
